# Regional and local factors interact to shape colonization and extinction dynamics of invasive *Hydrilla verticillata* in a patchy landscape

**DOI:** 10.1002/ece3.11558

**Published:** 2024-06-18

**Authors:** Joshua T. Armstrong, Lesley P. Bulluck, Andrew T. Davidson, Charles Ryland Stunkle, James R. Vonesh

**Affiliations:** ^1^ Department of Integrative Life Sciences Virginia Commonwealth University Richmond Virginia USA; ^2^ Center for Environmental Studies Virginia Commonwealth University Richmond Virginia USA

**Keywords:** dynamic occupancy models, flooding, *Hydrilla verticillata*, invasive species, rock pools

## Abstract

Understanding the response of species to global change requires disentangling the drivers of their distributions across landscapes. Colonization and extinction processes, shaped by the interplay of landscape‐level and local patch‐level factors, are key determinants of these distributions. However, disentangling the influence of these factors, when larger‐scale processes manifest at local scales, remains a challenge. We addressed this challenge by investigating the colonization and extinction dynamics of the aquatic plant, *Hydrilla verticillata*, in a complex riverine rock pool system. This system, with hundreds of rock pools experiencing varying flooding frequencies, provided a natural laboratory to examine how a single landscape‐level disturbance can differentially impact colonization and extinction depending on local patch characteristics to shape species distributions. Using 5 years of data across over 500 sites and more than 5000 surveys, we employed dynamic occupancy models to model colonization, extinction, and changes in *Hydrilla* patch occupancy while accounting for imperfect detection. Our results revealed that larger, infrequently flooded pools closer to the river were more likely to be colonized. In contrast, local extinction of Hydrilla was more likely in smaller pools closer to the river that flooded frequently. These findings underscore the importance of considering context‐dependence in species distribution models. The same landscape‐level disturbance (flooding) had opposing effects on colonization and extinction, with the direction and magnitude of these effects varying with local patch characteristics. Our study highlights the need for integrating local and landscape‐level factors, and considering how larger‐scale processes play out at the patch level, to understand the complex dynamics that shape species distributions.

## Graphical abstract

Using dynamic occupancy modeling, we explored drivers of colonization and extinction dynamics of an invasive species, *Hydrilla verticillata*, within a novel complex landscape of 500+ riverine rock pools. We found that landscape and patch level characteristic, along with disturbance via flooding, influenced the probabilities of both colonization and extinction. Larger, less frequently flooded pools had high colonization probabilities, while smaller, more frequently flooded pools had higher extinction probabilities.

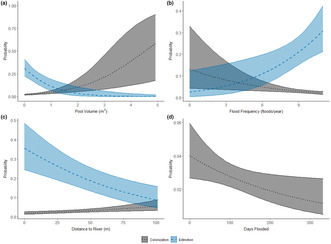

## 1 INTRODUCTION

Understanding how species respond to environmental change is a central challenge in ecology. Species distributions are inherently dynamic, fluctuating across space and time as a result of the interplay between colonization and extinction processes (Guisan & Thuiller, 2005). These processes are driven by a complex interaction of factors operating at both local (patch) and landscape scales, including habitat suitability, disturbance regimes, and biotic interactions (Honnay et al., 2005). In heterogeneous landscapes, where habitat patches vary in size, quality, and connectivity, understanding the drivers of patch colonization and extinction is essential for predicting species distributions (Hanski & Ovaskainen, 2003). This knowledge is particularly critical for developing effective conservation strategies, mitigating biodiversity loss, and managing both invasive and rare species (Fletcher & Fortin, 2018; Hanski, 2011; With, 2002).

The theory of island biogeography provides a basic framework for conceptualizing patch dynamics (MacArthur & Wilson, 1967). This theory posits that patch size and connectivity influence colonization and extinction rates, with larger, well‐connected patches exhibiting higher colonization and lower extinction probabilities (MacArthur & Wilson, 1967). Empirical studies in terrestrial systems have largely supported this theory, highlighting the importance of patch characteristics in shaping species distributions (Fahrig, 2003; Fukami, 2010).

However, disturbance regimes can also significantly influence colonization and extinction dynamics. Disturbances such as wildfires, floods, and hurricanes can act as drivers of local extinction, potentially eliminating populations from individual patches (Buckley et al., 2007; Catford et al., 2012; Sousa, 1979). Paradoxically, disturbances can also facilitate colonization, particularly for non‐native species, by disrupting established communities and creating opportunities for new arrivals (Lockwood et al., 2007). The frequency of disturbances can also increase some species colonization rates (Catford et al., 2012; Salemme & Fraterrigo, 2021). Taken as a whole, the effect of disturbance on colonization and extinction can have opposing effects on a population. For example, studies have shown that for lizard (*Anolis* spp.) and plant populations on Caribbean islands, hurricanes cause extinction events on some islands, while also facilitating colonization events on other islands (Morrison & Spiller, 2008; Schoener et al., 2001). In riverine systems, flooding can promote the colonization of some species, while leading to the extinction of others (Havel et al., 2000; Lafferty et al., 1999).

The dual role of disturbance as both a driver of extinction and a facilitator of colonization makes it challenging to predict its net effect on species occupancy. In some cases, disturbances may lead to population turnover within patches, with some populations going extinct while others colonize (e.g., Morrison & Spiller, 2008; Schoener et al., 2001), resulting in no net change in overall occupancy. Alternatively, disturbances may cause shifts in the relative importance of colonization and extinction, leading to changes in the underlying dynamics governing species distributions. Disentangling the complex interplay of patch characteristics and disturbance regimes is crucial for understanding how these factors shape species distributions. This is particularly challenging in systems where the same disturbance can have opposing effects on colonization and extinction, and where the magnitude and direction of these effects may vary depending on local patch characteristics.

In this study, we investigate the occupancy dynamics of the aquatic plant *Hydrilla verticillata* in a complex riverine rock pool system. This system, characterized by hundreds of rock pools experiencing varying levels of flooding disturbance, provides a unique opportunity to examine how landscape‐level processes (flooding) interact with local patch characteristics (pool size, depth, connectivity) to shape colonization and extinction dynamics. We expected that the net effect of flooding on *Hydrilla* occupancy will depend on the balance between its opposing effects on colonization (via dispersal) and extinction (via disturbance), with the relative importance of these effects varying across patches with different characteristics. We predicted that larger, infrequently flooded pools closer to the river channel (a potential source of colonists) will have higher colonization and lower extinction probabilities. By explicitly modeling colonization and extinction processes, we aim to disentangle the complex interactions between patch characteristics and disturbance regimes, and to provide insights into the mechanisms underlying species distributions in dynamic landscapes.

## 2 METHODS

### 2.1 Study system

**FIGURE 1:**
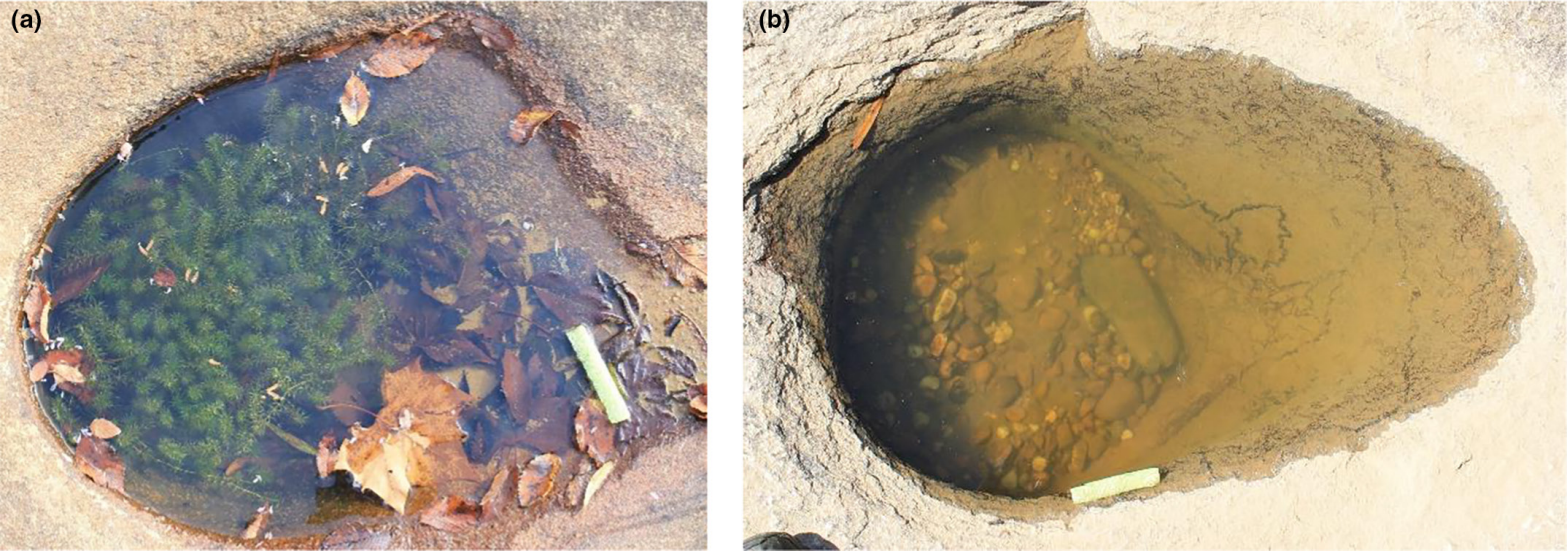
Rock pool with and without *Hydrilla*. This picture is an exemplar of what (a) *Hydrilla* looks like growing in a riverine rock pool in our system and (b) what a rock pool without *Hydrilla* looks like. The green pool noodle at the right of the pool is a 10 cm floating scale for reference.

**FIGURE 2:**
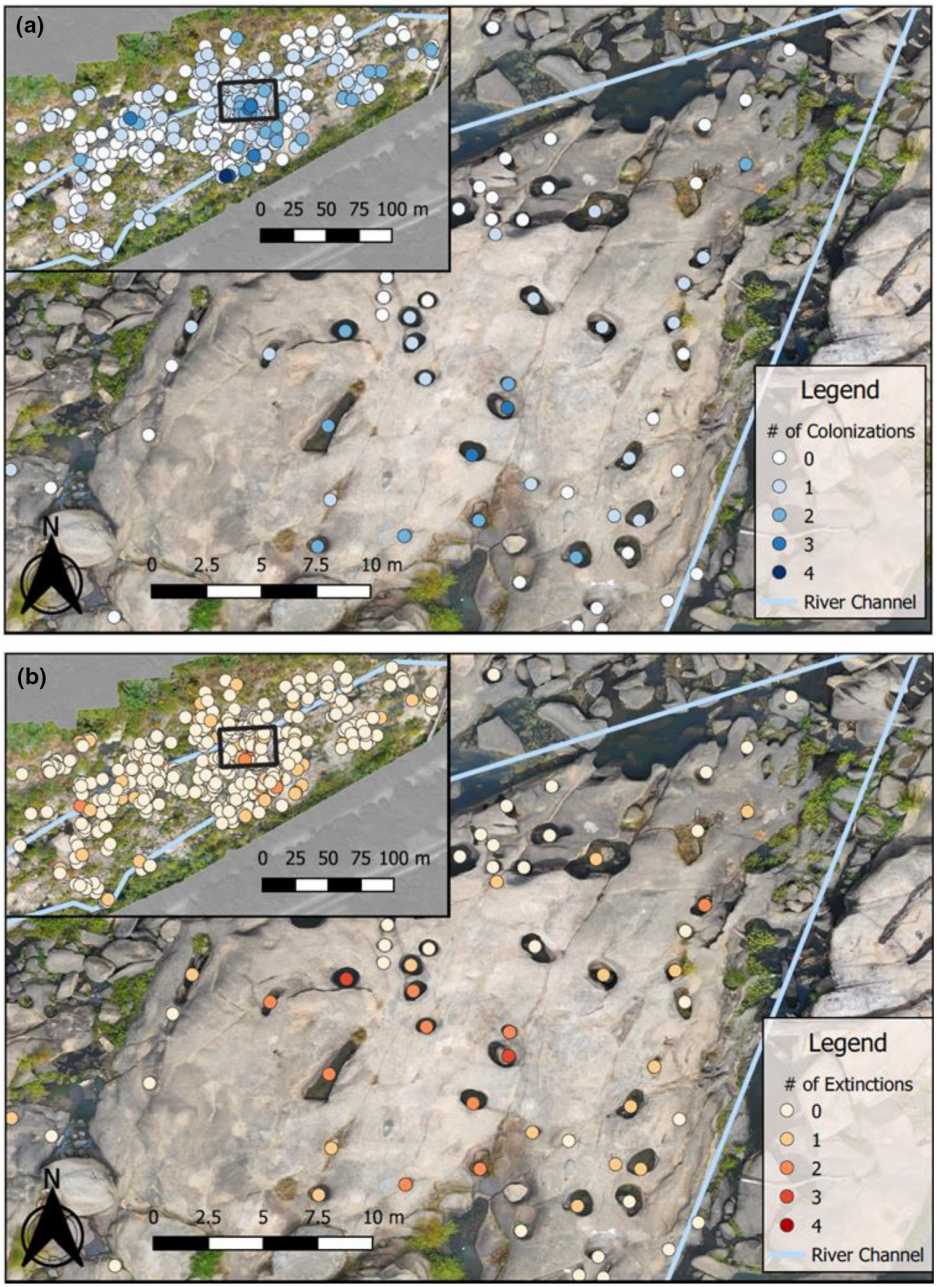
Map of *Hydrilla* colonizations and extinctions in the rock pool system from 2017 to 2021. These maps show (a) the number of colonization events that a pool experienced (blue), and (b) the number of extinction events that a pool experienced (red). All of the points on the map represent every rock pool sampled for this study. The blue line in each panel represents the most persistent river channel at the lowest river height (1.07 m) and within this map, flows from the left to the right. This reach of the river is located James River in Richmond, Virginia, USA, near Belle Isle (37°31′44.98″ N, 77°27′9.14″ W).

Our study site consists of a system of over 750 rock pools located on the James River in Richmond, Virginia near Belle Isle (37°31′44.98″ N, 77°27′9.14″ W; Figures 1 and 2). Rock pools are depressions that are formed in river bed rock through erosion and other geological processes (Pelletier et al., 2015) and are commonly inhabited by a range of different aquatic taxa, including plants, macroinvertebrates, and fish (Jocque et al., 2010). Rock pools can form a complex of aquatic communities, consisting of dozens to hundreds of pools (Brendonck et al., 2016; Jocque et al., 2010; Ren et al., 2016), and because of this, they are considered natural microcosms that offer ideal conditions to test ecological theories (Srivastava et al., 2004). With hundreds of individual pools potentially connected by water during flooding events, this system allows for robust investigation of colonization and extinction dynamics, and how they are influenced by ecosystem level processes and characteristics, such as disturbance frequency, patch size, and connectivity. The rock pool system tends to experience large floods periodically throughout the year that inundate a large proportion of the rock pools (Stunkle et al., 2021).

The system has been mapped and georeferenced using both aerial drone images (DJI Mavic Pro 2) and GPS (Leica GX1230). The size of individual rock pools is highly variable, but on average, pools are 304 ± 10.6 L (mean ± SE), with 3.0 ± 0.12 m (mean ± SE) distance separating individual pools. Previous work in this system has shown how dynamic and variable these rock pools are in regards to temperature (max. temp range 20–42°C during summer months) (Davidson et al., 2024), dissolved oxygen (1.54–9.43 mg/L, mean = 5.70 mg/L, *n* = 20, Richie Dang ‐ RHD), and pH (7.18–9.15, mean = 8.30, *n* = 55) (Jackson, 2010). The same can be said about the ecosystem level processes as well in regards to gross primary productivity (0–12.45 g/m^2^/d, mean = 2.68 g/m^2^/d, *n* = 20, Richie Dang ‐ RHD), particulate organic nitrogen (0.35–39.43 mgN/L, mean = 13.12 mgN/L, *n* = 20, Richie Dang ‐ RHD), and CHLa (2.7–1130.9 μg/L, mean = 218.8 μg/L, n = 20, Richie Dang ‐ RHD).

*Hydrilla verticillata* is a submerged aquatic macrophyte and pervasive invasive species in this system (Figure 1; hereafter *Hydrilla*; Royle, 1839). *Hydrilla* is native to warmer parts of Asia and has become a prolific invasive species in many aquatic habitats such as wetlands, ponds, rivers, and riverine rock pools across Europe, Africa, and both North and South America (Langeland, 1996). *Hydrilla* easily invades communities and rapidly grows and reproduces through asexual fragmentation (Langeland, 1996), which can be triggered by removal attempts and other disturbance (e.g., boating, and flooding). *Hydrilla* invasions have led to shifts in community composition resulting in lower species richness of both plants and animals (Barrientos & Allen, 2008; Gentilin et al., 2020; Posey et al., 1993). *Hydrilla* is not only found in the rock pools and the surrounding river, but is also found in many lakes and ponds throughout the eastern United States, including within the region where this study was conducted.

### 2.2 Data collection

Biotic and abiotic data were obtained for 506 randomly sampled rock pools (pools numbered and selected using a random number generator) between 2017 and 2021, from April through November, *Hydrilla's* growing season (True‐Meadows et al., 2016). While *Elodea canadensis* (confamilial to *Hydrilla*) is found within the James River system, we have not documented it in our rock pool system. To ensure that *Hydrilla* was properly identified, surveyors were trained to identify *Hydrilla*, specifically through serrated leaf margins and the number of whorls on its stem. *Hydrilla* occupancy (the presence or absence) was sampled by passively observing each rock pool for 1 min. Two to five repeat surveys were carried out opportunistically within a day of the first sample, when no flooding had occurred, by different surveyors; these surveys were used to estimate *Hydrilla* detection probability.

Patch size was defined by the pool depth and volume. Rock pool depth and volume were moderately correlated (*r* = 0.431), but we consider both here, because they likely influenced *Hydrilla* differently. Volume measures the space that *Hydrilla* has available to grow, while depth influences the amount of light it can obtain. Physical characteristics of the pools (e.g., depth, length, width) were measured in centimeters using a meter stick. Depth was estimated by averaging four measurements taken randomly throughout the pool. Length was measured as the longest distance across the pool, with width being measured perpendicular to the length. The volume (m^3^) for each pool was calculated using the equation for an elliptical cylinder (Equation 1).

(1) $$V=r_{1}r_{2}\mathrm{\pi h}$$

The volume (*V*) of the elliptical cylinder is equal to the first radius (*r*_1_) times the second radius (*r*_2_) times the height (*h*) times pi (*π*).

Connectivity was defined by the distance a pool was from the river channel and the distance a pool was from its nearest neighboring pool. Using the R packages *spatstat* (Version 3.0‐3; Baddeley & Turner, 2005), and *raster* (Version 3.6‐14; Hijmans, 2022), we calculated the nearest neighbor distance (m) and the distance a pool was from the river channel (m) by measuring the shortest distance from a pool to the most persistent river channel at the lowest river level observed, 1.07 m. Flooding was defined by the mean annual number of flood events per pool (flood frequency) and mean number of days a pool was flooded per year (days flooded) during our study, both of which were calculated for every pool in the system across several years (Stunkle et al., 2021). All of these variables allowed for a spatiotemporal analysis of *Hydrilla* dynamics.

### 2.3 Dynamic occupancy modeling

We used dynamic occupancy modeling (MacKenzie et al., 2003) to estimate the probability of occupancy, colonization, and extinction for *Hydrilla* in the rock pool system. This framework models temporal change in occupancy to investigate drivers behind colonization (i.e., a patch transitioning from being unoccupied to occupied by *Hydrilla*) and extinction (i.e., a patch transitioning from being occupied to unoccupied by *Hydrilla*) dynamics (MacKenzie et al., 2003). Unlike other occupancy models (Hanski, 1994, 1998; Moilanen, 1999), this framework takes into account imperfect detection of focal species (MacKenzie et al., 2003) which is a challenge inherent to occupancy approaches. This is because failing to observe a species at a patch may reflect a true absence of that species at the patch, but it could also occur if a surveyor merely failed to detect that species at the time the patch was surveyed (MacKenzie et al., 2003). By accounting for imperfect detection rates, the dynamic occupancy modeling framework allow for less biased estimates of colonization and extinction rates. This framework also does not make restrictive assumptions about perfect detection and process stationarity that incidence function models make (MacKenzie et al., 2003).

We used the *unmarked* package (Version 1.2.5) in R (Version 4.1.2), to hierarchically model detection first. Important detection predictors were then carried over to occupancy models (Fiske & Chandler, 2011; MacKenzie et al., 2017). There are three assumptions to these models: (1) replicate surveys at a site during a single season are independent, (2) occurrence state does not change over replicate surveys at site *i* during season *t*, (3) there are no false‐positive errors, i.e., a species can only be overlooked where it occurs, but it cannot be detected where it does not occur (MacKenzie et al., 2003). Within the rock pool system, flooding can occur any time leading to potential colonization/extinction events, thus, defining a season as a year violates the closure assumption (assumption 2). For this analysis, each sampling day was considered its own primary period (*N* = 33) with between 2 and 5 replicate surveys per pool happening within a day, resulting in five secondary sampling periods. Defining the seasons as such and accounting for detection probability adheres to the model assumptions that there is no change in occupancy state during a primary period.

We defined individual covariates as either site, observational, or yearly covariates, and standardized all continuous variables by expressing them as z‐scores. Observational covariates (i.e., covariates that change every observation) were applied only to detection models (MacKenzie et al., 2003) and included day of the year (date) and days since last sampling of the rock pool (days since). Across the entire length of the study, an average of 134 ± 59 days (mean ± SE) passed between sample days. However, this included the winter season (December through March) where no sampling occurred, and during the growing season the average time between sample days was only 44 ± 12 days (mean ± SE). Site covariates (i.e., covariates that are site specific and do not change over the course of the study) included pool depth, pool volume, flooding frequency, nearest neighbor, distance to river, and days flooded. Yearly covariates cannot be used to predict occupancy because occupancy is calculated from only the first year's data (MacKenzie et al., 2003). Sample year was the only yearly covariate (i.e., covariates that change each year) however due to primary periods not being defined by years, this was only included to assess whether detection was influenced by the year the rock pool was sampled.

Models for detection, occupancy, colonization, and extinction were compared using Akaike's information criterion (AIC). All models with ΔAIC < 2.0 were considered viable models and if there was more than one top performing model, we used model averaging to calculate parameter estimates (Burnham & Anderson, 2002a, 2002b). Detection was modeled first, with the covariates date, days since last sampled, pool depth, and pool volume. The most parsimonious top model was carried through to model occupancy, colonization, and extinction. Occupancy was then modeled, consisting of models with the covariates: pool depth, pool volume, flood frequency, distance to river, nearest neighbor, days flooded, and two interaction terms, volume interacting with flood frequency and volume interacting with days flooded. Both top occupancy and detection models was carried through to model colonization and extinction, which were modeled independently of each other, i.e., when modeling colonization, extinction was held constant and vice versa. We compared 11 models for detection and 24 models for occupancy, colonization, and extinction (See the Supporting Information for more details). Based on our predictions of how the *Hydrilla* dynamics are influenced by the system, we expected the top models for occupancy, colonization, and extinction to include predictors of pool size, connectivity, and flooding, with flooding being the strongest predictors.

### 2.4 Post‐hoc test

Due to this sampling scheme of only sampling during growing months, it is possible that we mischaracterized the senescence and regrowth of *Hydrilla* during the winter months as extinction and colonization events, respectively. Further, we could not directly assess this in our dynamic occupancy models because days since last sampling could only be included in the detection models and not colonization and extinction models. To assess the possibility of mischaracterizing senescence and regrowth as extinction and colonization, we ran post‐hoc analyses using logistic regression to explore whether the amount of time between sampling over the winter months had any impact on colonization and extinction. Due to the simplicity of these models, detection was not accounted for.

## 3 RESULTS

**FIGURE 3:**
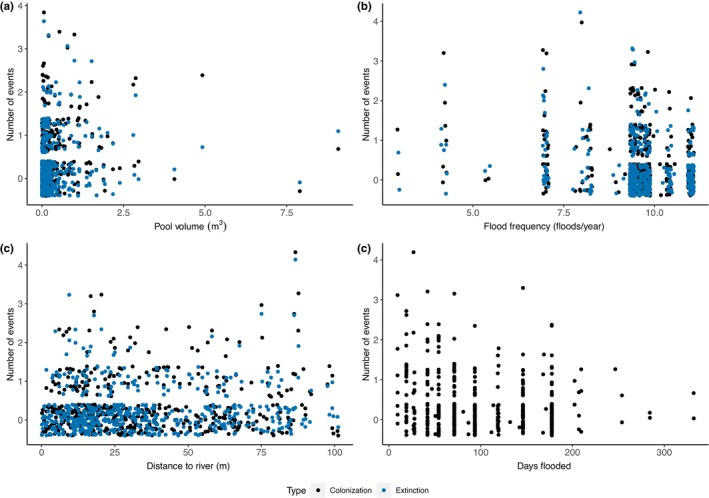
Raw number of colonization and extinction events. The raw numbers of *Hydrilla* colonization (black) and extinction (blue) events, over the entire study period, as a function of (a) pool volume, (b) flood frequency, (c) distance to river, and (d) days flooded. The data in all panels is jittered to better visualize the data.

Over the course of the study (2017–2021, over 5000 observations), *Hydrilla* was detected in 147 out of 506 pools, with a total of 133 colonization events and 55 extinction events (Figure 3). There were also 147 persistence events, where pools stayed occupied from one sample period to the next. Nearly all 506 rockpools were surveyed and more repeat surveys occurred during 2020 and 2021 resulting in more detections/non‐detections compared with 2017–2019 (Table 1).

**TABLE 1:** Yearly sampling effort and detections/non‐detections of *Hydrilla*.

Year	Pools sampled	Surveys conducted	Pools with repeat sampling	Detections	Non‐detections
2017	67	93	0	25	68
2018	211	356	1	20	335
2019	230	523	16	32	476
2020	506	1461	181	126	1005
2021	505	2051	121	148	1660

*Note*: Pools sampled are the total number of pools that were sampled each year. Surveys conducted is the total number of surveys that were carried out. Pools with repeat sampling are the number of pools that had secondary sampling conducted to assess detection. Detections are the total number of times *Hydrilla* was detected during a survey and non‐detections are when it was not.

### 3.1 Detection

There were four top models for *Hydrilla* detection which included the sampling date, days since last sampling, pool volume, and pool depth. The date was included in all four top models and, after model averaging, was the only covariate with a strong positive effect on detection, i.e., detection of *Hydrilla* is greater later in the year. Overall, detection probabilities increased from ~30% early in the growing season to ~98% later in the growing season. The other survey covariates (days since last sampling, pool volume, and pool depth) were not strong predictors of *Hydrilla* detection (i.e., parameter estimates include 0). We therefore included date as the only survey covariate for detection as we moved to the next steps of explaining variability in colonization and extinction.

### 3.2 Occupancy

There were two top models (ΔAIC = 1.87, top model *ĉ* = 1.47) for *Hydrilla* occupancy which included: pool depth, pool volume, flood frequency, and the interactions between pool volume and flood frequency (Table 2). The pool volume had a strong positive effect on *Hydrilla* occupancy (Table 3); larger pools had a greater probability of being occupied by *Hydrilla*, with pools >2 m^2^ having a 100% chance of being occupied. There was a significant interaction between volume and flood frequency for *Hydrilla* occupancy (Table 3). We found that flooding had a negative effect on *Hydrilla* occupancy in small pools and a positive effect in larger pools. Pool depth did not influence *Hydrilla* occupancy (Table 3). The best performing occupancy model included pool volume, flood frequency, and the interaction between the two; this model was carried through to model colonization and extinction.

**TABLE 2:** Top ranking models (ΔAIC ≤ 2.0) for *Hydrilla* occupancy, colonization, and extinction with AIC comparison values.

	Model	*n* Parameters	Negative log‐likelihood	AIC	ΔAIC	Weighted AIC
Occupancy	psi(volume + freq+volume:freq)gam(.)eps(.)p(date)	8	954.184	1924.369	0.000	0.364
	psi(depth + volume)gam(.)eps(.)p(date)	7	956.122	1926.244	1.875	0.143
Colonization	psi(volume + freq+volume:freq)gam(depth + volume + river + flooded + freq + volume:flooded + volume:freq)eps(.)p(date)	15	924.662	1879.324	0.000	0.565
Extinction	psi(volume + freq + volume:freq)gam(.)eps(depth + volume + river+flooded + freq)p(date)	13	930.778	1887.556	0.000	0.503

*Note*: Model covariates are: depth (pool depth – cm), volume (pool volume – m^3^), river (distance to river channel – m), freq (flood frequency – floods per year), flooded (days flooded – days). Occupancy is *psi*(), colonization is *gam*(), extinction is *eps*(), and detection is *p*().

**TABLE 3:** Model parameter estimates, standard error (SE), and 95% confidence intervals (CI).

	Predictor	Estimate	SE	Lower 95% CI	Upper 95% CI
Occupancy	Intercept	−1.27	0.37	−2.00	−0.54
	Depth	0.73	0.44	−0.14	1.60
	Volume	2.54	1.13	0.32	4.76
	Flood frequency	−0.26	0.27	−0.80	0.27
	Volume:flood Frequency	1.50	0.69	0.15	2.84
Colonization	Intercept	−3.57	0.13	−3.83	−3.32
	Depth	0.06	0.11	−0.16	0.28
	Volume	0.63	0.15	0.33	0.93
	Distance to river	0.28	0.09	0.10	0.46
	Distance to the nearest pool	−0.07	0.10	−0.26	0.13
	Days flooded	−0.22	0.10	−0.42	−0.03
	Flood frequency	−0.21	0.08	−0.35	−0.06
	Volume:days flooded	−0.31	0.16	−0.62	0.01
	Volume:flood frequency	0.20	0.14	−0.08	0.48
Extinction	Intercept	−1.20	0.20	−1.59	−0.80
	Depth	−0.04	0.14	−0.32	0.23
	Volume	−0.93	0.25	−1.42	−0.44
	Distance to river	−0.44	0.12	−0.67	−0.21
	Distance to the nearest pool	−0.09	0.13	−0.34	0.16
	Days flooded	0.15	0.13	−0.10	0.40
	Flood frequency	0.30	0.11	0.09	0.51

*Note*: These are values for the top performing model for occupancy, colonization, and extinction and are *z*‐transformed values. Occupancy shows the model averaged estimates of the top performing models. There was only one colonization and extinction model with ΔAIC < 2.0.

### 3.3 Colonization

**FIGURE 4:**
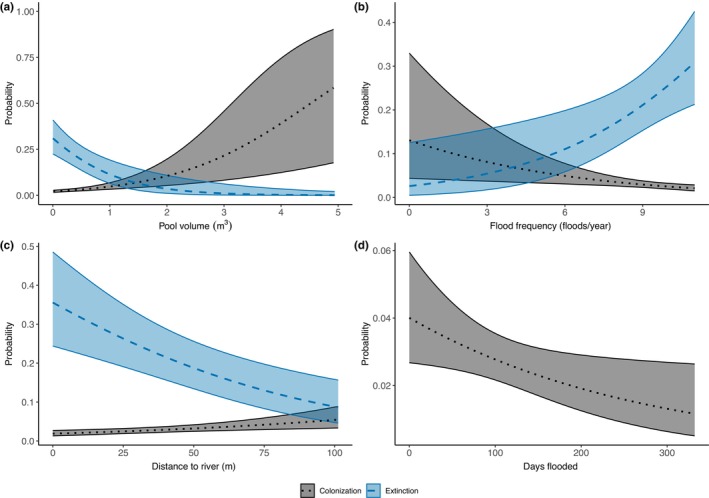
Colonization and extinction probabilities. The probability of *Hydrilla* colonization (gray), and extinction (blue) as a function of (a) pool volume, (b) flood frequency, (c) distance to river, and (d) days flooded. These predicted probabilities were estimated from the top performing model. 95% confidence intervals are shown.

**FIGURE 5:**
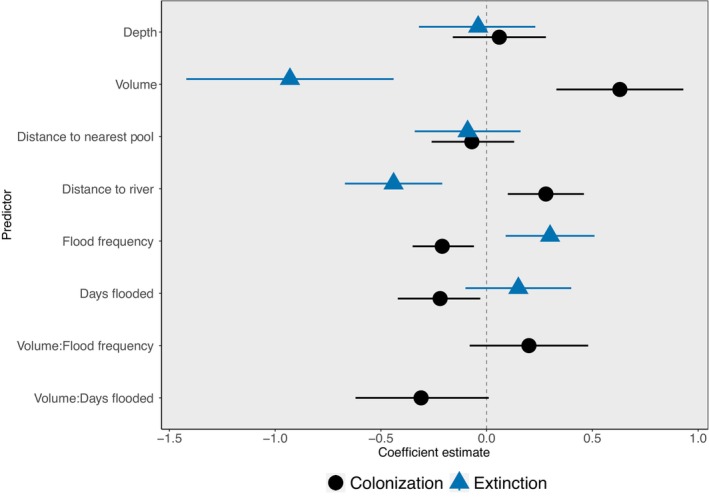
Effect size estimates for predictors of *Hydrilla* colonization (black) & extinction (blue). The predictors in these models were standardized using z‐score standardization. These estimates are the top performing model coefficient estimates and lines represent a 95% confidence interval.

Colonization reflects a transition from *Hydrilla* absence to presence between sampling events (*n* = 133). There was only one top model for colonization (AIC_w_ = 0.60, *ĉ* = 1.06), and it included pool depth, pool volume, distance to river channel, distance to nearest neighbor, flood frequency, days flooded, and the interactions between volume and flood frequency and volume and days flooded (Table 2). Pool volume had a positive effect on colonization (Table 3); larger pools had a greater probability of being colonized by *Hydrilla* (Figure 4a). Flood frequency had a negative effect on colonization (Table 3, Figure 4b). The distance from the river channel had a positive effect on colonization (Table 3); pools further from the river channel have a slightly higher probability of being colonized by *Hydrilla* (Figure 4c). The number of days a pool was flooded had a negative effect on *Hydrilla* colonization (Table 3); longer flooded periods lead to lower probability of colonization (Figure 4d). Despite being present in the top model, the distance a pool was from another pool and the interaction terms between pool volume and flooding frequency and duration did not affect the probability of colonization (i.e., the confidence intervals surrounded zero; Table 3 and Figure 5).

### 3.4 Extinction

Extinction reflects a transition from *Hydrilla* presence to absence between sampling events (*n* = 55). As with colonization, volume, distance to river, and flooding frequency influenced *Hydrilla* extinction, but the direction of the relationships was opposite (Figure 4). There was only one top model for extinction (AIC_w_ = 0.69, *ĉ* = 0.99) which included the covariates pool depth, pool volume, distance to the river channel, distance to nearest neighbor, flood frequency, and days flooded (Table 2). Pool volume had a negative effect on extinction (Table 3); smaller pools had a higher probability of extinction (Figure 4a). Flood frequency had a positive effect on extinction (Table 3); as flood frequency increased the extinction probability increased (Figure 4b). The distance a pool was located from the river channel had a negative effect on extinction (Table 3); the closer a pool was to the river channel, the higher the extinction probability (Figure 4c). The depth of the pool, the distance from another pool, and number of days flooded had no effect on extinction (Table 3 and Figure 5). Thus, because the probability of Hydrilla remaining present in a pool can be estimated as 1—extinction, this means *Hydrilla* was more likely to persist in larger pools that were further from the river channel and flooded less frequently.

### 3.5 Post‐hoc test

Based on these results of our post‐hoc test, ~18% (*N* = 10) of extinction events and ~7% (*N* = 10) of colonization events could have been senescence and regrowth. We found that the time between samples both within the year (colonization: Est. = 0.22, SE = 0.20, *p*‐val. = .26; extinction: Est. = 0.54, SE = 0.32, *p*‐val. = .09) and between years, during winter (colonization: Est. = 0.16, SE = 0.19, *p*‐val. = .39; extinction: Est. = 0.20, SE = 0.32, *p*‐val. = .53), did not significantly predict colonization nor extinction. These results suggest that any transitions during this time may not necessarily be senescence and regrowth, but true colonization and extinction events. Furthermore, we are confident in our results because we were only sampling during the growing season, not these winter months.

## 4 DISCUSSION

Our study investigated the colonization and extinction dynamics of the invasive aquatic macrophyte, *H. verticillata*, in a complex riverine rock pool system, revealing the intricate interplay of patch characteristics, landscape features, and disturbance regimes in shaping species distributions. We found that both local (pool volume) and landscape‐level (distance to the river) factors, in conjunction with the disturbance regime (flooding), significantly influenced the colonization and extinction probabilities of *Hydrilla*. These findings highlight the importance of considering context‐dependent effects and the interplay of multiple factors in understanding species dynamics in heterogeneous landscapes.

Larger pools, further from the river and less frequently flooded, exhibited higher colonization probabilities for *Hydrilla*. This result aligns with the predictions of the theory of island biogeography (MacArthur & Wilson, 1967). Our findings also echo previous empirical studies that have demonstrated the positive effect of patch size on colonization for both native and invasive taxa (Crooks et al., 2001; Fedrowitz et al., 2012; Lenda et al., 2010). Larger pools may offer more stable habitats, with greater resource availability and reduced environmental stress, thereby facilitating the establishment of new populations.

Interestingly, we found a negative association between colonization probability and proximity to the river, contrary to the expectation that connectivity to a potential source population would enhance colonization (Fedrowitz et al., 2012, Lenda et al., 2010). This suggests that the river may not be the primary source of *Hydrilla* propagules in this system, and that other colonization mechanisms, such as dispersal within the rock pool network or via waterfowl, may be more important. It is also possible that flooding, while potentially increasing connectivity, may act as a disturbance that inhibits the establishment of new populations in frequently inundated pools. Alternatively, *Hydrilla* turions (dormant buds capable of growing new plants) could be another mode of dispersal, rather than fragmentation of vegetative material during flooding events; they have been shown to exhibit intermediate to long distance dispersal and withstand large disturbances (Madsen & Smith, 1999). Turions could be dispersed during large floods that connect occupied *Hydrilla* pools to unoccupied pools. However, due to the dormancy of these turions within the sediment layer, it is possible that colonization events could occur and not be observed because turions are difficult to detect. While we did not explore the presence of other plants, larger pools anecdotally tend to have more plant richness, and this could have facilitated colonization if *Hydrilla* propagules became stuck in established plant stems/roots (Capers et al., 2007). This highlights the complex and potentially opposing effects of disturbance on colonization, and the need to consider multiple factors simultaneously when interpreting occupancy patterns.

The probability of *Hydrilla* extinction was higher in smaller, frequently flooded pools closer to the river. This pattern is consistent with basic theory, which predicts higher extinction rates in smaller (MacArthur & Wilson, 1967), a prediction that has since been observed in empirical studies (Crooks et al., 2001; Lenda et al., 2010). Additionally, our findings align with previous *Hydrilla* research demonstrating the negative impacts of disturbance on species persistence (Sousa, 2011). In our system, flooding likely acts as a disturbance, dislodging *Hydrilla* from smaller, shallower pools closer to the river where hydraulic forces are stronger. The increased susceptibility of smaller pools to extinction may also be attributed to their reduced resource availability and greater vulnerability to environmental stress. Furthermore, the relationship between pool volume and extinction probability may be influenced by differences in hydrologic processes. Larger, deeper pools may experience weaker hydraulic forces during flooding events, reducing the likelihood of *Hydrilla* being washed out (Pelletier et al., 2015). The increased water volume in larger pools may also buffer against extreme temperature fluctuations and other environmental stressors, further contributing to their lower extinction rates.

Our results challenge the prevailing notion that disturbance invariably promotes invasion. While disturbance can create opportunities for invasive species by disrupting established communities and increasing resource availability (Lockwood et al., 2007), it can also act as a stressor that limits population growth and increases extinction risk (Catford et al., 2012; Gerhardt & Collinge, 2003; Hobbs & Huenneke, 1992; Lake & Leishman, 2004; Marchetti et al., 2004; Minchinton, 2002; With, 2004). However, there is an alternative hypothesis, the passenger model, that posits that disturbance has no effect on the invasive population (MacDougall & Turkington, 2005). In our study system, flooding appears to have a dual role, potentially facilitating colonization in some patches while driving extinction in others. The opposing effects of disturbance on colonization and extinction underscore the importance of considering context‐dependence in reference to a species' distribution. The net effect of a disturbance on species occupancy will depend on the balance between its positive and negative influences, which can vary depending on patch characteristics, disturbance intensity and frequency, and species‐specific traits. In the case of *Hydrilla*, flooding appears to primarily act as a disturbance, increasing local extinction in smaller, more vulnerable pools, while its role in facilitating colonization may be limited.

The observed patterns of colonization and extinction suggest the presence of source‐sink dynamics in the rock pool system. Larger, less disturbed pools may act as sources of *Hydrilla* propagules, while smaller, frequently disturbed pools may act as sinks, with populations maintained primarily through immigration rather than local reproduction. Source‐sink dynamics occur when many subpopulations of a species exist in habitat patches that may not be suitable for the species (i.e. sinks) but their landscape‐level persistence is maintained through some populations existing in suitable habitat and dispersing to other patches (i.e., sources) (Pulliam, 1988). There are few examples of how invasive species may exhibit source‐sink dynamics and include taxa such as mussels, amphibians, and fish (Bobeldyk et al., 2005; Dauphinais et al., 2018; Sepulveda, 2018). This has important implications for managing the spread of *Hydrilla*, as targeting the removal of large source populations may be a more effective strategy than attempting to control the species in numerous small, ephemeral sink populations (Sepulveda, 2018).

While our study focused on the influence of flooding, patch size, and connectivity, other factors such as water chemistry and biotic interactions may also play a role in *Hydrilla* colonization and extinction dynamics. It is widely known that the water chemistry of an aquatic system can heavily influence the distribution of macrophytes (Jeppesen et al., 2007; Spence, 1967). While these rock pools are a fraction of the size of even a small pond, they still have dynamic chemical cycling and the water chemistry of the pools falls within the range of *Hydrilla's* tolerance (Sousa, 2011; Spence, 1967; Steward & Van, 1987; Yang et al., 2008). Therefore, in this system, the water chemistry of the pools may not have a significant impact on the colonization and extinction dynamics of *Hydrilla*, but due to the dynamic nature of these pools, water chemistry likely rapidly fluctuates, which has been shown to influence *Hydrilla* invasion in other aquatic systems (Salgado et al., 2023). Future research should investigate these additional factors to gain a more comprehensive understanding of the mechanisms shaping species distributions in dynamic landscapes, however obtaining regular water chemistry data for all 506 rock pools was not feasible and beyond the scope of this study.

A potential weakness of our study is that one of the assumptions of this modeling framework is that there are no false positives, i.e., a species found in a patch that is not actually present. With our study system of rock pools, we assumed the same assumption. *Hydrilla* is the only submerged macrophyte in this system with its distinct morphology. While we trained surveyors on how to identify *Hydrilla*, there is another species of submerged macrophyte located in the James River system that looks similar, *Elodea canadensis*. While it has yet to be documented within this rock pool system, there is always a possibility that in has gotten into the system and was misidentified as *Hydrilla*. In our case, this was unlikely, yet not impossible. Others have improved this modeling framework in order to account for false positive, particularly in taxa where it is more common such as birds and amphibians (Royle & Link, 2006). Future studies in similar systems to ours would benefit from using these models if false positives are thought to be an issue; not doing so could alter parameter estimates.

Extrapolating results to other scales or systems has been a persistent problem in ecology. Our results align with what others have found in other systems and across spatial scales. Although there are some things to consider when translating these results across scales and systems. Populations across complex landscapes have been shown to be impacted differently depending on the configuration of the landscape (Fagan, 2002). Scale has been shown to greatly impact occupancy, colonization and extinction (Frey et al., 2012; Stevens & Conway, 2020) and we advise against the extrapolation of our results to other spatial scales and systems (Nordén et al., 2020). Regardless, *Hydrilla* can be found in many different types of aquatic systems, such as lakes, rivers, and ephemeral ponds, and our results are likely applicable to other rock pool and ephemeral pond systems where invasive macrophytes exist and flooding is common.

Our findings also have broader implications for understanding the response of species to global change. As climate change alters disturbance regimes and environmental conditions, understanding how species respond to these changes will be critical for predicting future species distributions and developing effective conservation strategies. Our study emphasizes the importance of considering context‐dependence and the interplay of local and landscape‐level factors in understanding species responses to environmental change. By disentangling the complex interactions between these factors, we can gain valuable insights into the mechanisms underlying species persistence in dynamic landscapes and develop more effective strategies for managing invasive species and conserving biodiversity.

## AUTHOR CONTRIBUTIONS

**Joshua T. Armstrong:** Conceptualization (lead); data curation (lead); formal analysis (lead); investigation (equal); methodology (lead); software (lead); visualization (lead); writing – original draft (lead); writing – review and editing (equal). **Lesley P. Bulluck:** Conceptualization (supporting); software (supporting); supervision (equal); writing – review and editing (equal). **Andrew T. Davidson:** Investigation (supporting); writing – review and editing (equal). **Charles Ryland Stunkle:** Investigation (supporting); writing – review and editing (equal). **James R. Vonesh:** Conceptualization (supporting); formal analysis (supporting); investigation (supporting); supervision (equal); writing – review and editing (equal).

## CONFLICT OF INTEREST STATEMENT

Authors have no conflicts of interest.

## Supporting information


Data S1.

## Data Availability

The data that support the findings of this study are openly available in Dryad at https://doi.org/10.5061/dryad.jsxksn0fn.
