# Supplementary material for: Regional and local factors interact to shape colonization and extinction dynamics of invasive Hydrilla verticillata in a patchy landscape
Source: Ecol Evol. 2024 Jun 18;14(6):e11558. doi: 10.1002/ece3.11558 (PMC11184213; doi:10.1002/ece3.11558)
Supplement: Supplementary file 1 — Data S1. [file ECE3-14-e11558-s001.docx]

**TABLES**

**Detection:**

**Table S1.** All tested models for *Hydrilla* detection with AIC comparison values. Model covariates tested are: date (Julian day), depth (pool depth – cm), days (days since last sampling), and volume (pool volume – m^3^). The model *p()* shows the detection model being tested.


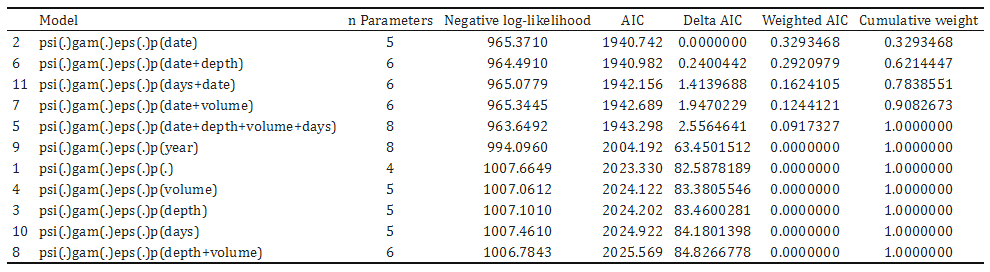


**Table S2.** Model summary for the top performing detection models (ΔAIC < 2.0). All statistics were obtained from model averaging.


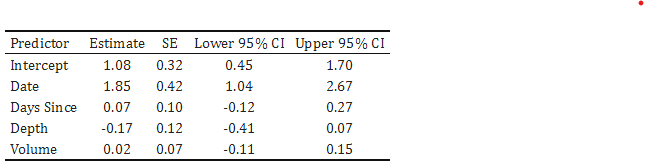


**Occupancy:**

**Table S3.** All tested models for *Hydrilla* occupancy with AIC comparison values. Model covariates tested are: depth (pool depth – cm), volume (pool volume – m^3^), river (distance to river channel – m), nn_dist (distance to nearest pool – m), freq (flood frequency – floods per year), flooded (days flooded – days). The model *psi()* shows the occupancy model being tested.


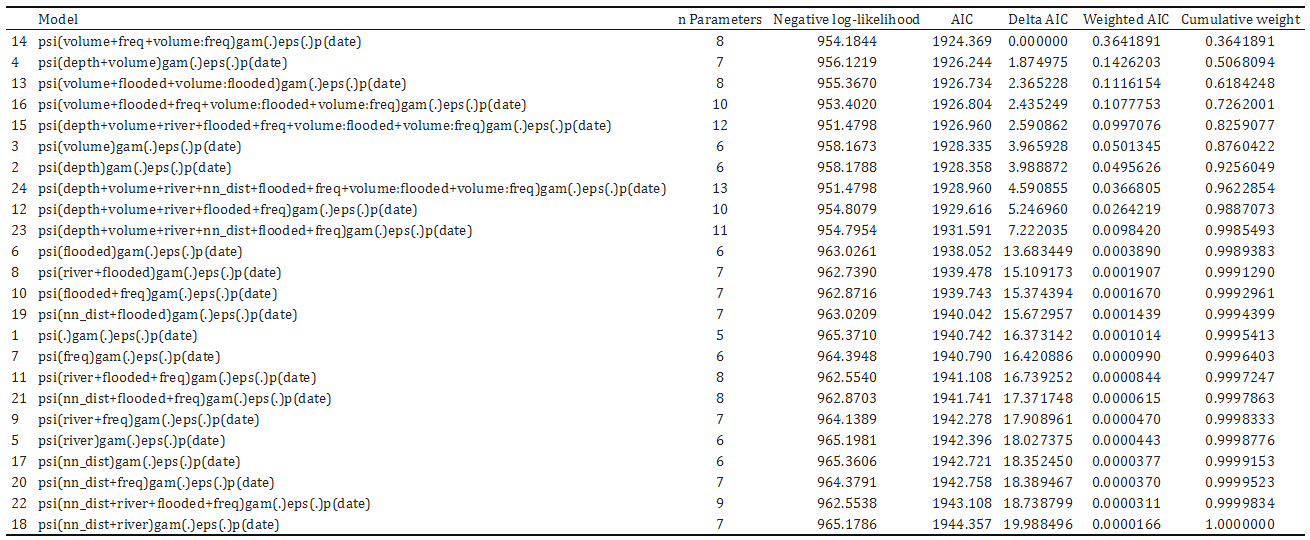


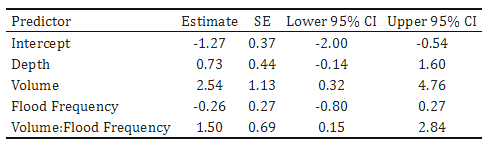
**Table S4.** Model summary for the top performing occupancy models (ΔAIC < 2.0). All statistics were obtained from model averaging.

**Colonization:**

**Table S5.** All tested models for *Hydrilla* colonization with AIC comparison values. Model covariates tested are: depth (pool depth – cm), volume (pool volume – m^3^), river (distance to river channel – m), nn_dist (distance to nearest pool – m), freq (flood frequency – floods per year), flooded (days flooded – days). The model *gam()* shows the colonization model being tested.

**
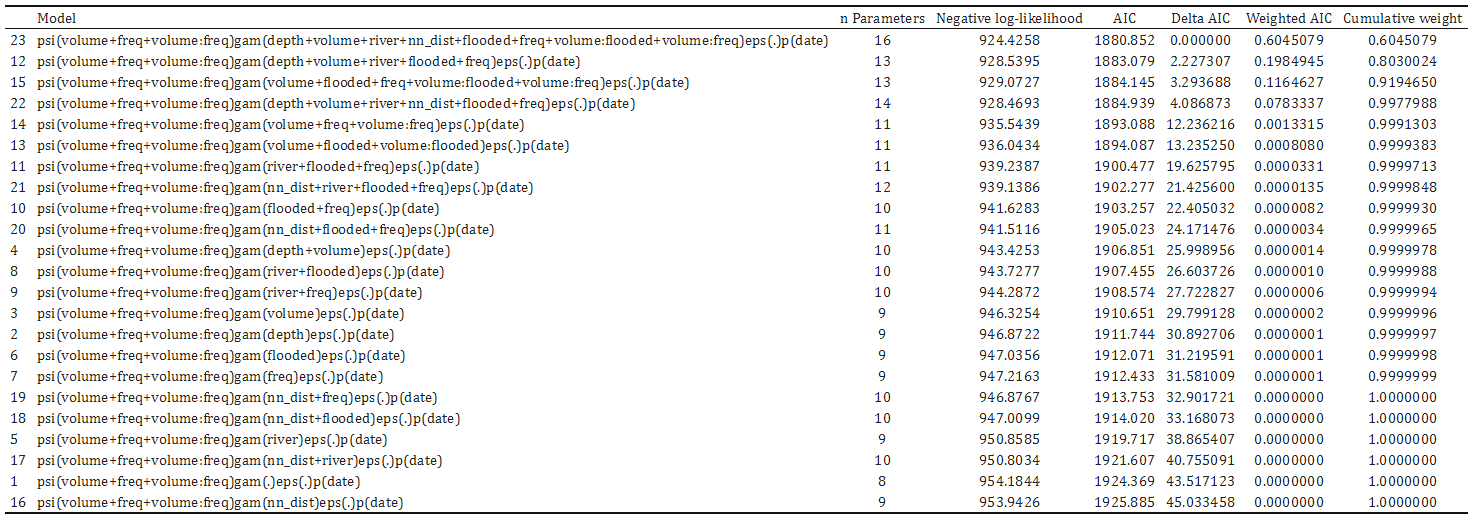
**

**Table S6.** Model summary for the top performing colonization model (ΔAIC < 2.0).

**
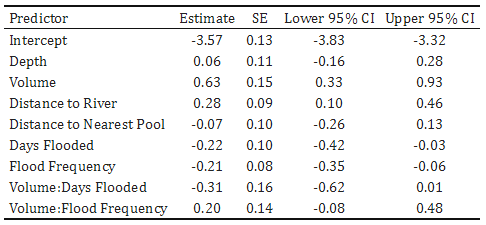
**

**Extinction:**

**Table S7.** All tested models for *Hydrilla* extinction with AIC comparison values. Model covariates tested are: depth (pool depth – cm), volume (pool volume – m^3^), river (distance to river channel – m), nn_dist (distance to nearest pool – m), freq (flood frequency – floods per year), flooded (days flooded – days). The model *eps()* shows the extinction model being tested.

**
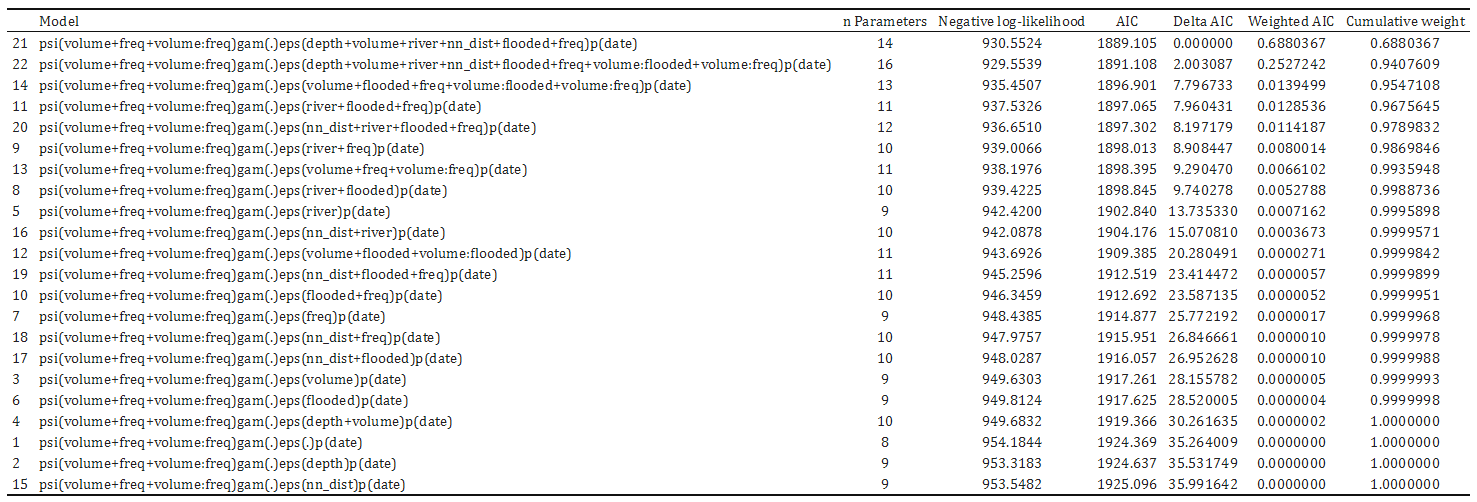
**

**Table S8.** Model summary for the top performing extinction model (ΔAIC < 2.0).


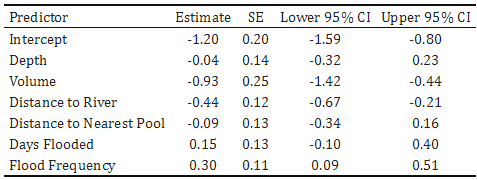


**EXTRA INFORMATION**

**Transition events: Colonization events = 133 & Extinction events = 55**

**Detection gof test results:**

Goodness-of-fit for dynamic occupancy model

Number of seasons: 33

Chi-square statistic:

Season 1 Season 2 Season 3 Season 4 Season 5 Season 6 Season 7 Season 8 Season 9 Season 10

0.2824 11.4277 4.3947 25.7297 0.0249 2.5322 1.6435 1.5227 2.4714 0.1512

Season 11 Season 12 Season 13 Season 14 Season 15 Season 16 Season 17 Season 18 Season 19 Season 20

4.7906 0.4366 14.5663 1.3535 0.4519 5.1402 0.5188 0.0016 2.6553 20.4817

Season 21 Season 22 Season 23 Season 24 Season 25 Season 26 Season 27 Season 28 Season 29 Season 30

10.0244 16.0869 0.5296 1.9580 0.0329 4.2300 302.5224 31.9171 71.4120 6.8702

Season 31 Season 32 Season 33

0.1407 0.1047 0.1119

Total chi-square = 546.5174

Number of bootstrap samples = 100

P-value = 0.04

Quantiles of bootstrapped statistics:

0% 25% 50% 75% 100%

107 184 237 299 1183

Estimate of c-hat = 2.04

**Occupancy gof test results:**

Goodness-of-fit for dynamic occupancy model

Number of seasons: 33

Chi-square statistic:

Season 1 Season 2 Season 3 Season 4 Season 5 Season 6 Season 7 Season 8 Season 9 Season 10

0.0032 4.9438 0.4100 29.5549 0.0458 2.3317 1.3533 1.3451 2.9508 0.1384

Season 11 Season 12 Season 13 Season 14 Season 15 Season 16 Season 17 Season 18 Season 19 Season 20

5.3112 0.4793 16.5796 1.0733 0.3340 5.4170 0.7137 0.0047 1.9805 39.2889

Season 21 Season 22 Season 23 Season 24 Season 25 Season 26 Season 27 Season 28 Season 29 Season 30

8.1508 19.7934 0.5290 1.7660 0.0067 3.9055 211.5099 32.0430 61.5118 7.6992

Season 31 Season 32 Season 33

0.1648 0.1059 0.1126

Total chi-square = 461.5576

Number of bootstrap samples = 100

P-value = 0.14

Quantiles of bootstrapped statistics:

0% 25% 50% 75% 100%

121 212 276 400 728

Estimate of c-hat = 1.47

**Colonization gof test results:**

Goodness-of-fit for dynamic occupancy model

Number of seasons: 33

Chi-square statistic:

Season 1 Season 2 Season 3 Season 4 Season 5 Season 6 Season 7 Season 8 Season 9 Season 10

0.1787 5.6942 0.6378 24.3182 0.1590 2.3767 1.3354 1.3806 4.1388 0.0973

Season 11 Season 12 Season 13 Season 14 Season 15 Season 16 Season 17 Season 18 Season 19 Season 20

2.7887 0.4806 7.8673 1.0397 0.2442 4.5445 0.6161 0.1868 0.4465 21.7694

Season 21 Season 22 Season 23 Season 24 Season 25 Season 26 Season 27 Season 28 Season 29 Season 30

8.0827 16.6060 0.4838 0.7332 0.4655 3.7964 66.2449 25.8347 79.1083 5.7539

Season 31 Season 32 Season 33

0.1538 0.4053 0.4340

Total chi-square = 288.4028

Number of bootstrap samples = 100

P-value = 0.3

Quantiles of bootstrapped statistics:

0% 25% 50% 75% 100%

129 192 254 297 817

Estimate of c-hat = 1.06

**Extinction gof test results:**

Goodness-of-fit for dynamic occupancy model

Number of seasons: 33

Chi-square statistic:

Season 1 Season 2 Season 3 Season 4 Season 5 Season 6 Season 7 Season 8 Season 9 Season 10

0.0051 8.2948 0.7636 24.1014 0.0002 1.4942 1.1273 1.1190 5.4868 0.0832

Season 11 Season 12 Season 13 Season 14 Season 15 Season 16 Season 17 Season 18 Season 19 Season 20

3.2112 0.6012 9.2015 1.0563 0.2843 4.5336 1.0522 0.3941 0.1934 34.1361

Season 21 Season 22 Season 23 Season 24 Season 25 Season 26 Season 27 Season 28 Season 29 Season 30

8.6947 19.8766 0.3723 0.4646 0.7544 3.4909 72.9862 28.0778 63.1741 6.5021

Season 31 Season 32 Season 33

0.2411 0.3959 0.4258

Total chi-square = 302.5959

Number of bootstrap samples = 100

P-value = 0.27

Quantiles of bootstrapped statistics:

0% 25% 50% 75% 100%

106 215 266 307 1270

Estimate of c-hat = 0.99
